# Transient Sideroblastic Anemia Post-COVID-19 Infection

**DOI:** 10.7759/cureus.30275

**Published:** 2022-10-13

**Authors:** Nikhil Mukhi, Luis R Soto, Aparna Vuppala

**Affiliations:** 1 Hematology/Oncology, St. Francis Hospital, Tulsa, USA; 2 Pathology, St. Francis Hospital, Tulsa, USA; 3 Medicine, St. Francis Hospital, Tulsa, USA

**Keywords:** covid-19, myeloproliferative neoplasm disease, myelodysplastic disease, post-covid-19 conditions, reticulocytopenia, sideroblastic anemia

## Abstract

A 57-year-old gentleman presented to the hospital with progressive fatigue and dyspnea on exertion three months after recovering from COVID-19. He was noted to have severe anemia with reticulocytopenia. After excluding vitamin deficiencies and heavy metal toxicities, a bone marrow aspirate and biopsy were performed, which showed erythroid predominant trilineage maturing hematopoiesis with 79% ring sideroblasts and no dysplasia. SF3B1 mutation was negative. He was diagnosed with sideroblastic anemia and became transfusion-dependent. He was treated with an erythropoiesis-stimulating agent and luspatercept with transient improvement in anemia. After 12 months of treatment, anemia spontaneously improved. Repeat bone marrow biopsy showed hypercellular marrow with 39% ringed sideroblasts. We suspect that this possibly was a delayed manifestation of COVID-19 infection.

## Introduction

Sideroblastic anemia (SA) is a group of blood disorders characterized by ineffective erythropoiesis and accumulation of ringed sideroblasts (RSs) in bone marrow [1]. The accumulation of iron-laden mitochondrial complexes encircling the erythroblast nucleoli is the hallmark feature of RS. Initially described in the 1940s, the last 30 years have seen a seismic shift in our understanding of genetic abnormalities in the pathogenesis of RS. Next-generation sequencing has helped us find pathognomonic genetic mutations in two-thirds of SA cases. It can be congenital or acquired [2].

Congenital sideroblastic anemias (CSAs) are inherited mitochondrial disorders affecting heme synthesis. Germline mutations in the *ALAS2*, *SLC25A38*, and *SLC19A2* genes, have been implicated in its pathogenesis [2,3]. They can occur independently or can be syndromic, namely, Pearson marrow-pancreas syndrome. These patients almost exclusively present at a young age with microcytic/macrocytic/normocytic anemia and are transfusion-dependent [3].

Acquired sideroblastic anemia (ASA) is discovered in individuals over 40 years of age as incidental anemia. It is subcategorized into reversible SA from environmental factors and clonal stem cell disorder classified within the umbrella of myelodysplastic disease (MDS). Somatic mutations in the SF3B1 gene (a gene involved in RNA splicing machinery) have been reported to be particularly common (60-80%) in patients with SA [4]. Toxic/metabolic reversible SA cases have been attributed to copper deficiency, excessive alcohol use, and likely isoniazid and linezolid [5,6]. These are rare, and their prevalence is not known.

COVID-19 is a global pandemic manifesting as a respiratory illness. It has been associated with a variety of hematologic manifestations. The most frequent correlations have been seen with anemia and thrombocytopenia. Anemia has been attributed to inflammation and is transient. Rare cases of autoimmune hemolytic anemia and aplastic anemia have been defined in the literature [7,8]. So far, in clinical literature, no deleterious effects on hematopoiesis have been described.

We now discuss a case of SA three months after COVID-19 infection that spontaneously resolved after 15 months.

## Case presentation

A 57-year-old male presented to the hospital with complaints of progressive fatigue and dyspnea on minimal exertion, which had worsened for three months post-COVID-19 infection. He denied fever, chills, night sweats, or weight loss. His infection manifested as fever, cough, phlegm production, fatigue, and body aches. It was self-limiting and resolved without medications in five days. He had a long-standing history of diabetes, hypertension, and depression. His home medications included metformin, lisinopril, and sertraline. Medications had not been changed in several years. He denied alcohol use or illicit drugs. On physical examination, he was noted to have tachycardia without signs of lymphadenopathy or hepatosplenomegaly. Laboratory studies were significant for hypoproliferative microcytic anemia with normal kidney and liver function (Table 1).

**Table 1:** Laboratory values at presentation and 12 months later BUN, blood urea nitrogen; AST, aspartate transaminase; ALT, alanine transaminase

Lab test	At presentation	12 months later	Reference range
White blood cell count	11.3	8.7	4.6-12.4 k/cmm
Hemoglobin	7.7	13.7	12.8-17.4 g/dL
Mean corpuscular volume	65.6	82.2	80-100 cmic
Red cell distribution width	25.7	20.3	10.5-14.1%
Absolute reticulocyte count	9.2	34.2	17-9-111.2 K/cmm
Platelets	381	326	150-330 K/cmm
BUN	12	15	5-25 mg/dL
Creatinine	0.85	0.90	0.73-1.25 mg/dL
Calcium	8.3	9.3	8.5-10.7 mg/dL
AST	22	19	8-42 U/L
ALT	38	20	7-40 U/L
Albumin	3.6	4.1	3.4-4.7 mg/dL
Total bilirubin	0.5	0.4	0.1-1.2 mg/dL

Workup for anemia was negative for vitamin deficiencies, heavy metal toxicities, or hemolysis (Table 2). The stool for occult blood was negative. CT scan of the chest, abdomen, and pelvis was normal.

**Table 2:** Laboratory investigations for anemia LDH, lactate dehydrogenase; ANA, antinuclear antibody; RBC, red blood cell

Lab values	Results	Reference range
LDH	253	117-278 U/L
Copper	98.9	70-140 ug/dL
Zinc	70.5	60-120 ug/dL
Ferritin	1,127	10-259 ng/mL
Vitamin B12	479	213-816 pg/mL
Pyridoxine	13	5-50 ug/L
ANA	Negative	Negative
Parvovirus B19 PCR	Negative	Negative
Haptoglobin	315	22-200 mg/dL
RBC folate	1240	>366 ng/dL
Lead	Undetectable	<5 ug/dL

**Figure 1:**
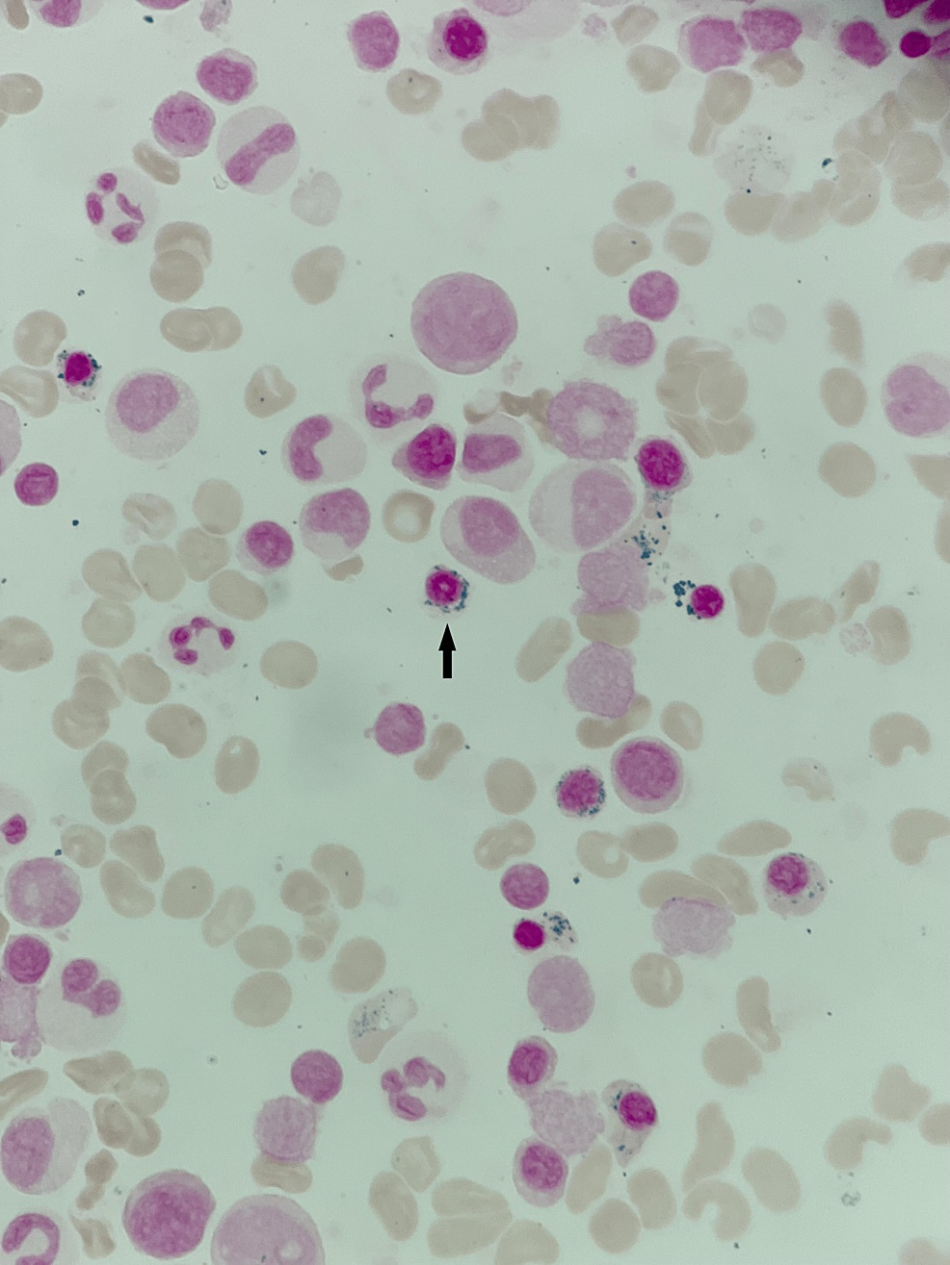
Prussian blue staining on bone marrow aspirate showing iron granules (blue) around a late erythroblast (magnification 1000x).

**Figure 2:**
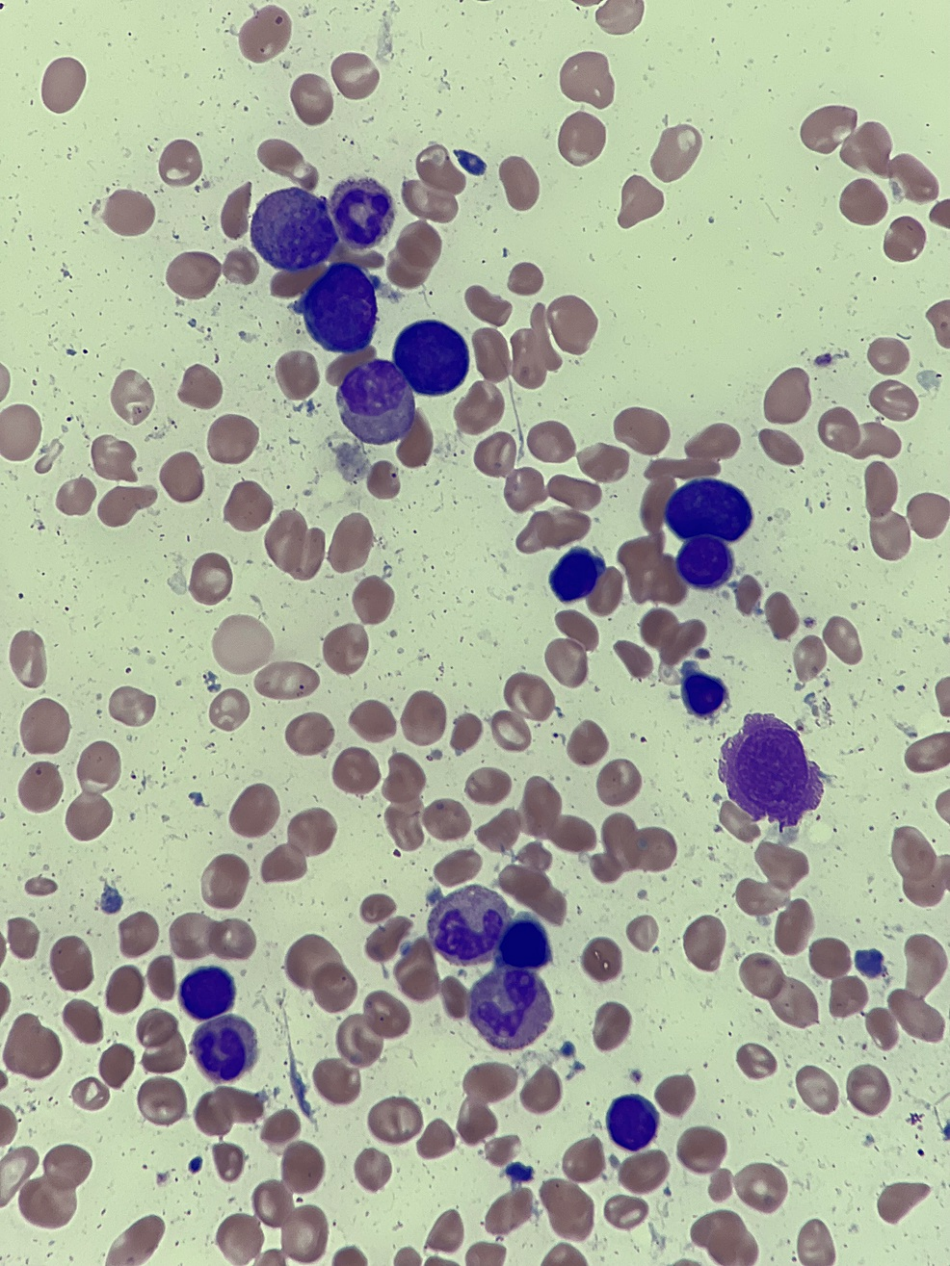
May Grunwald-Giemsa stain on bone marrow showing erythroblasts with megaloblastic changes in varying stages of maturation with no signs of dyserythropoiesis (magnification 1000x).

Bone marrow aspirate and biopsy showed 80-90% cellularity with erythroid predominant trilineage maturing hematopoiesis with 79% ring sideroblasts and no increase in blasts (Figure 1). No dysplastic features were noted (Figure 2). Karyotype was 46, XY (19)/47, XY, +Y (1). Next-generation sequencing was negative for *SF3B1* mutation and *TP53* mutation. Clinically, the patient did not fit the criteria for MDS and was diagnosed with anemia with RSs.

The patient’s hemoglobin (Hb) was lower than 8 gm/dL, and he required weekly blood transfusions. He was initiated on darbepoetin subcutaneously to reduce transfusion requirements. Darbepoetin was escalated to 200 mcg weekly with a goal Hb of more than 8 gm/dL. He had transitory response to therapy with improvement in Hb to 10 gm/dL before dropping to less than 8 gm/dL at the seven-month mark. He was subsequently switched to luspatercept, which was escalated to 1.75 mg/kg every 21 days. Four months into this therapy, the patient’s Hb improved to 13 gm/dL. His luspatercept was hence stopped. Four months have passed since his last dose, and his complete blood count remains normal (Table 1). Bone marrow biopsy shows hypercellular marrow with 39% RSs without significant dysplasia.

## Discussion

Severe acute respiratory syndrome coronavirus 2 (SARS-CoV-2) is an infective-inflammatory disease caused by the novel COVID-19 virus. It has infected 612 million people worldwide, with over 6.5 million deaths as of September 2022, making it a global pandemic. The symptoms can vary from asymptomatic disease to severe acute respiratory distress syndrome. A wide variety of hematologic manifestations, including lymphopenia, anemia, thrombocytopenia, elevated ferritin, coagulopathy, and elevated D-dimers, have been associated with the disease. Pathogenesis is attributed to marked elevation in pro-inflammatory markers such as IL-1β, IL-2, IL-4, IL-6, IL-10, TNF-α, and IFNγ, causing an exaggerated immune response [9]. The inflammatory response causes iron dysmetabolism, leading to increased hepcidin levels, reduced iron utilization, elevated ferritin level, and anemia [10]. These manifestations are seen in active COVID-19 infection and improve after the resolution of the infection. Delayed manifestations are being increasingly described in the literature [11]. So far, to our knowledge, long-term effects on the hematopoietic system have not been reported.

Our case describes the first case of delayed SA post-COVID-19. The presence of reticulocytopenia and pathognomonic RSs in bone marrow confirmed the diagnosis. The absence of clonality and dysplasia in bone marrow biopsy goes against the diagnosis of MDS. Acquired vitamin deficiencies and heavy metal toxicities were ruled out.

The underlying pathophysiology of SA is ineffective erythropoiesis. This can occur as defects in heme biosynthesis, iron-sulfur cluster assembly, or impaired synthesis of mitochondrial or cytosolic proteins necessary for heme synthesis. These mechanisms lead to the build-up of iron-laden mitochondrial complexes around the erythroblast nucleoli rather than the standard incorporation of iron into protoporphyrin IX (PPIX) in the mitochondrion [12]. We postulate in our patient that COVID-19 infection likely caused an immune-mediated genetic defect in a hematopoietic clone, leading to ineffective erythropoiesis and the development of RSs. Comprehensive genomic profiling did not show any genetic alterations. We hypothesize that COVID-19 likely caused a genetic alteration in a novel gene causing SA. A recent meta-analysis showed elevated ferritin (8%) and IL-6 levels (3%) several months after COVID-19 recovery [11]. We suspect that after the resolution of the inflammatory process, the altered hematopoietic cell diminished, as noted in the bone marrow.

This is the first case describing the effects of COVID-19 on hematopoietic stem cells; we anticipate that other effects will become more evident over time.

## Conclusions

ASA is seen in older patients with ineffective erythropoiesis. It occurs due to the acquisition of mutations in hematopoietic stem and progenitor cells and reversible factors such as heavy metal toxicities. The literature does not show infectious pathology as the underlying cause of this disease. However, the novel COVID-19 virus causes an infective-inflammatory illness, and its long-term effects on hematopoietic progenitor cells are yet to be discovered. Our case favors an association between the infection and the development of SA; however, more research is warranted to confirm the causal relationship.
